# Euglycemic Diabetic Ketoacidosis in a Pregnant Woman With Severe COVID-19: A Case Report

**DOI:** 10.7759/cureus.21649

**Published:** 2022-01-26

**Authors:** Megan L Harman, Emery M Cuellar, Allyson R Burkhart Denora, Megha Pokhriyal, Saad Mussarat

**Affiliations:** 1 Internal Medicine, Eastern Virginia Medical School, Norfolk, USA; 2 Internal Medicine, Naval Medical Center Portsmouth, Portsmouth, USA

**Keywords:** hypoxemic respiratory failure, euglycemic, diabetic ketoacidosis, pregnancy, covid-19

## Abstract

Euglycemic diabetic ketoacidosis (DKA) of pregnancy is an uncommon but serious condition that poses a substantial risk to the fetus. The physiological state of pregnancy itself predisposes women to ketosis and ketoacidosis, which can be further exacerbated by acute stressors such as infection. In this article, we describe a case of a pregnant woman with gestational diabetes and coronavirus disease 2019 (COVID-19) requiring mechanical ventilation who developed euglycemic DKA during her hospital course. Despite treating the patient with standard DKA protocol, fetal heart monitoring was non-reassuring and, hence, a cesarean section was performed. Postoperatively, her DKA resolved; however, she was maintained on supportive ventilation for continued management of her severe COVID-19 infection. In light of the ongoing pandemic, it is essential that healthcare teams closely monitor pregnant women presenting with COVID-19 infection for early signs of euglycemic DKA so that treatment may be initiated early and feto-maternal complications are avoided.

## Introduction

Euglycemic diabetic ketoacidosis (DKA) in pregnancy is an obstetric emergency that can cause substantial morbidity and even maternal and fetal demise if not identified and addressed early [1]. Even when both parties survive the acute event, fetal exposure to the prolonged state of acidosis can lead to devastating chronic neurological issues such as hypotonia, developmental delay, and seizure disorders [2]. Pregnancy in itself is well documented as a diabetogenic state. In particular, placental-derived hormones such as human placental lactogen confer relative resistance to insulin, leading to increased lipolysis and ketogenesis and ultimately increasing the risk of ketosis and ketoacidosis compared to the non-pregnant state [3]. 

Since the coronavirus disease 2019 (COVID-19) pandemic arose in late 2019, many researchers have evaluated the effects of COVID-19 in pregnancy and have found that maternal infection is associated with higher rates of preterm delivery, low birth weights, and stillbirth [4]. One study cited that as high as 91% of pregnant women that contracted COVID-19 were delivered by cesarean section prematurely [5]. In addition to the risk posed to pregnancy, COVID-19 has also been shown to facilitate fat breakdown, leading to an increased risk of ketosis and ketoacidosis among infected patients regardless of diabetes or pregnancy status [6]. As aforementioned, pregnant women are at baseline more susceptible to ketosis, so those who additionally contract COVID-19 are at even higher risk for ketoacidosis. Comorbid diabetes mellitus or gestational diabetes would further elevate the risk for ketoacidosis and associated complications. 

In a previous report by Pikovsky et al, two cases of euglycemic ketoacidosis in two near-term pregnant women with mild COVID-19 disease requiring only supplemental oxygen and medical management are discussed [7]. Here, we will discuss a case of euglycemic DKA in a pregnant woman at 28 weeks gestation with severe COVID-19 requiring mechanical ventilation. We will additionally discuss the importance of prevention and early recognition of euglycemic DKA in pregnant women, especially in those with concomitant COVID-19 infection. 

## Case presentation

The patient is a 32-year-old Caucasian female, gravida 2 para 0, at 28 weeks and three days gestation who presented to the emergency department with worsening symptoms of fatigue, congestion, shortness of breath, chills, wheezing, and chest tightness after testing positive for COVID-19 five days earlier. She was unvaccinated. Her medical history was significant for obesity and well-controlled gestational diabetes with a hemoglobin A1C of 6.0%, managed with insulin.

On admission, her vitals were notable for a heart rate of 107 beats per minute (60-100bpm), blood pressure of 118/78 (<120/80), respiratory rate of 28 breaths per minute (12-20), and O2 saturation of 98% on room air (95-100). Pertinent labs are highlighted in Table 1. Her chest x-ray was remarkable for mild patchy mid and lower pulmonary opacities suspicious for COVID-19 pneumonia. Computed tomography angiography (CTA) performed in the emergency department ruled out pulmonary embolism and showed multifocal peripheral pulmonary consolidation, a high confidence feature for COVID-19 pneumonia. She was initially managed with intravenous fluids, albuterol, dexamethasone, and therapeutic Lovenox. 

**Table 1:** Blood results on admission and at the time of onset of euglycemic DKA DKA: diabetic ketoacidosis

Parameter	Admission	At Time of DKA	Reference Value
White cell count (x 10^3/uL)	6.0	7.9	4.0-11.0
Hemoglobin (g/dL)	11.9	11.6	11.7-15.5
Hematocrit (%)	36.9	34.0	35.1-46.5
Fibrinogen (mg/dL)	509	-	200-425
Glucose (mg/dL)	96	174	70-99
Urea Nitrogen (mg/dL)	8	6	6-22
Creatinine (mg/dL)	0.6	0.4	0.5-1.2
Sodium (mmol/L)	133	136	133-145
Potassium (mmol/L)	4.4	4.0	3.5-5.5
Bicarbonate (mmol/L)	13	8	20-32
Lactate Dehydrogenase (U/L)	184	210	98-192
Anion Gap (mmol/L)	18	21	3-15
Beta Hydroxybutyrate (mg/dL)	-	41.9	0.2-2.8
Lactic Acid (mmol/L)	-	1.2	0.5-2.0
pH	7.329	7.289	7.35-7.45
pCO2 (mmHg)	22.6	15.2	34.0-45.0
PO2 (mmHg)	90.0	78.0	80-100
Base Excess (mmol/L)	-14.0	-19.0	-2.0-2.0
D-Dimer mg/L	1.27	0.67	0.0-1.12
Ferritin (ng/mL)	545	486	10-291

On the second day of admission, the patient became tachypneic and tachycardic with increased work of breathing, saturating at 94% on 2L nasal cannula. An arterial blood gas analysis (ABG) revealed pH 7.29 (7.35-7.45), pCO2 15.2 (34.0-45.0), pO2 78 (80-100), and HCO3 8 (20-32). Her labs were notable for blood glucose of 117 (70-99), beta-hydroxybutyrate 41.9 (0.2-2.8), and anion gap of 21 (3-15) indicating the development of an anion gap metabolic acidosis. Further labs are highlighted in Table 1. In the setting of normal lactic acid and blood urea nitrogen (BUN) levels, no history of alcohol abuse, and no prior use of drugs known to cause an anion gap metabolic acidosis, euglycemic DKA was determined to be the most likely explanation for her constellation of signs and symptoms and she was subsequently transferred to the ICU for management. She was started on an IV insulin drip and hydrated with lactated ringer's solution, and her electrolytes were repleted accordingly. 

On day four, her ICU course was complicated by severe hypoxic respiratory failure requiring mechanical ventilation. Fetal monitoring showed a non-reassuring fetal heart tracing with minimal to absent variability and recurrent late decelerations, so the decision was made to perform a primary low-transverse emergency cesarean section (C-section). Appearance, Pulse, Grimace, Activity, and Respiration (APGAR) scores were significantly depressed with 1 at one minute, 2 at five minutes, and 4 at 10 minutes, and so the baby was intubated. The patient’s anion gap had not closed after 48-72 hours on IV insulin drip and she was switched to weight-based dosing with 0.05 units/hour and supplemental D5.

On day five, the anion gap closed and her insulin requirements decreased, indicating resolution of the euglycemic DKA and gestational diabetes following her C-section. She remained intubated for another five days and was then successfully extubated. Sixteen days after her initial presentation, she was discharged home on oxygen with orders for home healthcare and outpatient pulmonary rehabilitation. On follow-up at three months post-hospitalization, she was still experiencing easy fatigability and “brain fog.” Her daughter was able to go home at 36 weeks after spending seven weeks in the neonatal intensive care unit (NICU) and reached her expected birth weight by her due date.

## Discussion

**Figure 1:**
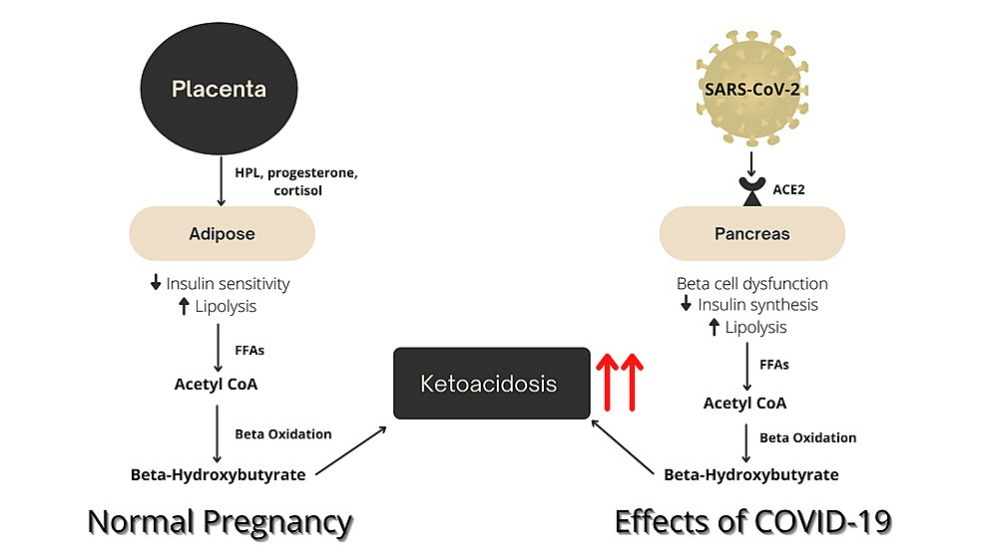
Physiology of normal pregnancy and effects of COVID-19 on ketogenesis COVID-19: coronavirus disease 2019; FFAs: free fatty acids; ACE2: angiotensin-converting enzyme 2; Acetyl CoA: acetyl coenzyme A; SARS-CoV-2: severe acute respiratory syndrome coronavirus 2; HPL: human placental lactogen

In normal pregnancy, the female body transitions to a diabetogenic state in order to facilitate the shunting of glucose across the placenta to the fetus for proper development. This change occurs in two primary ways. First, pancreatic beta cells undergo hyperplasia in order to secrete more insulin early in pregnancy [8]. This hyperplasia takes place in preparation for the second and third trimesters, during which insulin resistance is brought on by the inhibitory effect of placental hormones on the insulin sensitivity of peripheral tissues [9]. Despite insulin resistance, blood glucose levels in pregnancy remain low or normal due to factors such as increased glycogen storage, increased uptake by the fetoplacental unit, and increased peripheral usage [9]. This relatively hypoglycemic state causes the body to increase lipolysis as a fuel source for the pregnant mother. As a result, there is a concomitant increase in ketone formation via ketogenesis using the products of fat breakdown [10]. In the setting of stressors such as severe vomiting, fasting, or infection, ketogenesis may become further accelerated, predisposing women to the development of ketoacidosis [11]. This physiology is demonstrated in Figure 1. 

In light of the ongoing COVID-19 pandemic, it is important to consider the role COVID-19 may play in pregnancy. Even in those who are not pregnant and not diabetic, COVID-19 infection has been shown to be independently associated with accelerated ketogenesis and increased risk of ketoacidosis. A study by Li et. al. showed that among 658 patients hospitalized with confirmed COVID-19, 42 (6.4%) presented with ketosis and only 15 of them were diabetic. Of the 15 diabetic patients with ketosis, three (20%) developed diabetic ketoacidosis. Of the 27 non-diabetic patients with ketosis, two (7.4%) developed ketoacidosis. The researchers concluded that COVID-19 caused ketosis and ketoacidosis, and was additionally associated with longer hospital stays and increased mortality [6]. 

Though the mechanism underlying this association is not yet fully understood, it might have something to do with the interaction of the COVID-19 virus with the angiotensin-converting enzyme II (ACE2) receptor, which is expressed in the pancreatic islets, vascular endothelium, and adipose tissue [12]. The binding of the virus to the ACE2 receptor on pancreatic beta cells facilitates entry into the cells and may induce inflammation that contributes to the destruction of beta cells and their ability to produce or respond to insulin [13]. In this sense, COVID-19 infection may create an environment of pseudo-starvation leading to increased lipolysis, ketogenesis, and ultimately ketoacidosis (see Figure 1). This effect on metabolism is further demonstrated by the data showing that patients with diabetes who contract COVID-19 consistently experience worse outcomes than those without diabetes. One meta-analysis showed that the odds ratio for admission of diabetic patients with COVID-19 to the intensive care unit was 2.2 [14]. Furthermore, diabetes was twice as prevalent among those who died from COVID-19 compared to those who did not die from COVID-19 [14].

When ketoacidosis develops in the pregnant woman, complications may rapidly ensue if treatment is not initiated early. Euglycemic DKA in pregnancy is associated with recurrent decelerations, fetal hypoxia, and increased neonatal mortality [15]. For those who undergo a successful delivery, postpartum complications may still arise in the infant secondary to the elevated ketone body content that crossed the placenta in utero. These primarily are neurological problems including intellectual disability, structural abnormalities such as cleft lip, cleft palate, and neural tube defects [16], and, in rare cases, a devastating condition known as encephalomalacia [2]. Developmental, IQ, and motor proficiency scores in offspring exhibit an inverse relationship with maternal beta-hydroxybutyrate content in utero; the higher the ketone content in the mother, the lower the scores of the child [16]. Because fetal mortality is high and a strong linear relationship exists between maternal ketone content and poor outcomes postpartum, it is essential to identify and treat euglycemic DKA as early as possible. 

While the effects of maternal euglycemic DKA on the fetus are well documented, very little research has highlighted the specific complications that women experience during and after their hospitalization. However, physical complications of euglycemic DKA in pregnancy are comparable to those of general DKA. For example, one study describes numerous cardiovascular conditions precipitated by DKA including cardiac arrhythmias secondary to electrolyte imbalances, cardiogenic shock precipitated by acidosis, and even myocardial infarction [17]. Perhaps even more importantly, and even less often discussed, is the psychosocial and emotional impact of maternal DKA. Intubation can be distressing for both patients and their families and may be required in certain cases complicated by respiratory failure. Patients who clinically deteriorate might need to undergo an emergency C-section, which may have been undesired and is itself associated with sequelae such as infection, prolonged recovery, and scars that serve as a physical reminder of their trauma. These women must cope with the loss of a child or, if both survive delivery, the difficult reality that their baby may not lead a normal, healthy life. Furthermore, prolonged hospital stays can significantly alter a patient's ability to return to normal life after discharge. For instance, the patient discussed in this case report reported significant fatigue and "brain fog" lasting months after her hospitalization, necessitating that she put her education on hold. 

We propose to improve the early identification of euglycemic DKA in light of the COVID-19 pandemic by equipping pregnant women with the knowledge necessary to recognize the signs and understand the importance of early treatment. In addition to increased education, the American College of Obstetricians and Gynecologists (ACOG) and the Society for Maternal-Fetal Medicine (SMFM) recommend that all pregnant and lactating women be vaccinated against COVID-19 in order to prevent severe complications, such as euglycemic DKA [18]. It is vital that providers discuss and recommend vaccination against COVID-19 with pregnant patients, as vaccination during pregnancy has been proven to be safe [19]. While it is important to provide patients with the right educational tools, it is just as essential to ensure that medical staff is properly equipped to recognize and treat euglycemic DKA in pregnancy. Any pregnant woman presenting with severe nausea, hyperemesis, and/or abdominal pain [20], especially in the setting of concomitant COVID-19, should be promptly evaluated for euglycemic DKA. Additional signs of tachycardia, tachypnea, dehydration, or prior medical history of diabetes mellitus or gestational diabetes should further raise a red flag for ketoacidosis. If euglycemic DKA is suspected, healthcare providers should be vigilant with monitoring, treating, and potentially delivering the fetus if indicated to prevent fatal outcomes.

## Conclusions

Euglycemic DKA in pregnancy is a rare but serious medical condition associated with poor health outcomes. Early diagnosis may be facilitated by providing education focused on recognizing the signs and symptoms of euglycemic DKA to both patients and healthcare teams. Even in the absence of other risk factors like diabetes or pregnancy, patients presenting with COVID-19 are more susceptible to developing euglycemic DKA. It is therefore essential that healthcare providers discuss with their patients the importance of vaccination against COVID-19, particularly in those who are pregnant. With an end to the COVID-19 pandemic not yet in sight, physicians must take care to be vigilant when caring for pregnant women in the hospital setting. Early identification and prompt treatment of euglycemic DKA, especially among those with concomitant COVID-19 infection, is crucial in preventing maternal and fetal morbidity and mortality. 
